# A volitional account of aesthetic experience

**DOI:** 10.3389/fpsyg.2024.1480304

**Published:** 2024-10-24

**Authors:** Robert R. McCrae

**Affiliations:** Independent Scientist, Gloucester, MA, United States

**Keywords:** disinterested engagement, artistic devices, aesthetic qualities, Openness to Experience, volition

## Abstract

Aesthetic experience is an altered state of consciousness characterized by a detached absorption in an aesthetic object; it is a pleasant—sometimes ecstatic—liberation from the self and its agenda. I briefly review perceptual-cognitive and affective approaches used by psychologists to understand the phenomenon and suggest the need for a volitional perspective. To illustrate the nature and scope of aesthetic experience, I discuss nine varieties, elicited by different qualities in objects and evoking distinctive responses in perceivers. Over centuries, aesthetic devices have been developed that induce the aesthetic state by manipulating such psychological mechanisms as attention, appraisal, and empathy. I propose explanations for how several important devices operate, and why they are particularly effective in individuals high in the personality trait of Openness to Experience.

## Introduction

**FIGURE 1:**
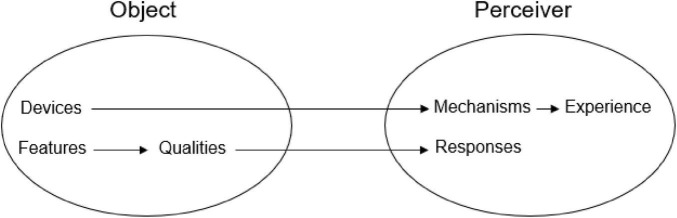
Schematic representation of the processes that induce aesthetic experience.

The aesthetic object—sonnet, sonata, sunset—has aesthetic devices which operate through psychological mechanisms such as attention, surprise, and identification to create a particular mental state we call the aesthetic experience. The object also has distinctive features—structure, complexity, tone color—that give it specific aesthetic qualities: We may find a poem to be elegant, a fugue intriguing, a view of nature overpowering. These qualities evoke corresponding aesthetic responses, including admiration, fascination, and awe. These relations are summarized in Figure 1.

A complete system of aesthetics would catalog the major devices used in each artform and explain the mechanisms that make the perceiver’s experience qualitatively aesthetic rather than ordinary. It would enumerate the types of qualities and show how each feature imparts a given quality to an object. It would also account for individual differences in aesthetic responses. Here I attempt to illustrate how such a system might be realized.

### Disclaimers

The aesthetic is often yoked with art, but this essay is narrowly focused on aesthetic experience from a psychological perspective and ignores the moral, spiritual, political, educational, and economic aspects of art. Most of the examples I will use are drawn from the arts, but art need not be aesthetic, and non-art often is.

I am not concerned here with criticism. One might substitute *Critic* for *Perceiver* in an alternative Figure 1, and fill it with such content as comprehension, analysis, evaluation, and emotional response (satisfaction, boredom, disgust)—but that is another topic. I make no effort to discover principles that distinguish good art from bad, or great from good. One individual may have the same aesthetic response to a greeting card verse that another has to Keats’ odes, and from the perspective taken here, these responses are equivalent.

Some writers reserve the term “aesthetic experience” for a kind of peak experience, equating it with “amazement, awe, elevation, and marvel” (Carroll, 2012, p. 168). Here I adopt a much lower threshold for designating an experience as aesthetic; enjoying a joke or admiring good penmanship can be mild and transient forms (cf. Whitfield and de Destefani, 2011).

Finally, I am not concerned with artistic creation or its psychology. The Greeks attributed artistic inspiration to the Muses; psychoanalysts regarded it as an expression of unconscious conflicts (Adorno, 1997). The beauty of the orchid is presumably the result of Darwinian evolution, and the music and literature of the future is likely to be generated by AI. How and why an aesthetic object was produced is not germane here; I am only concerned with the effects it produces in the perceiver and the means of producing those effects.

### Overview

I begin with a brief review of the psychological literature on aesthetics, contrasting the volitional perspective with the more familiar cognitive and affective perspectives. I then give a preliminary taxonomy of aesthetic qualities, which serves to illustrate the nature and wide scope of the topic. Next, I consider aesthetic devices and the corresponding psychological mechanisms that create the altered state of aesthetic experience. Given the relative paucity of empirical literature on this topic, the treatment is speculative, but it might stimulate the formulation of testable hypotheses. Finally, I touch on individual differences and argue that the personality trait of Openness to Experience facilitates the operation of the psychological mechanisms that produce the aesthetic state.

## Cognitive, affective, and volitional frames

In 1980, Hilgard (1980) provided a review of the venerable division of mental processes into the cognitive, affective, and conative (or volitional), and concluded that “cognitive psychology is ascendant at present” (p. 115). Since that time, increasing attention has been paid to emotion (Cowen et al., 2019; Frijda, 2007; Russell, 1980), but volition has been slighted. This is particularly evident in the psychology of aesthetics, where a long tradition of experimental aesthetics has been conceptualized in contemporary cognitive terms (e.g., Brielmann and Dayan, 2022; Pelowski et al., 2016), and where several new analyses of emotional responses to art and beauty have been offered (Juslin, 2013; Tan, 2000; Menninghaus et al., 2019). The present essay draws attention to volitional issues.

### Perceptual-cognitive approaches

The psychological study of aesthetics traces its origins to Fechner’s (1876) research on preferences for the proportions of rectangles, and was relaunched a century later by Berlyne (1974). Since that time, hundreds of articles have reported experiments in which respondents were briefly presented with (usually visual) stimuli and asked to assess their beauty or indicate how much they liked them. In a widely cited review, Reber et al. (2004) argued that a single principle accounted for most of the findings: The features found in stimuli judged to be more attractive—such as symmetry, familiarity, clarity, and prototypicality—all lead to more fluent processing, where fluency is essentially “the ease of identifying the physical identity of the stimulus” (p. 367). Fluent processing is intrinsically pleasant, and perceivers attribute this pleasure to the object and therefore judge it to be beautiful.

That review many be an accurate interpretation of the bulk of studies of empirical aesthetics, but to anyone outside the tradition, it seems curiously simplistic. Even researchers within the tradition have proposed that there are aesthetic effects beyond mere pleasure. Armstrong and Detweiler-Bedell (2008) argued that the pleasure aroused by fluent images is a mild sense of relief that our concepts of the world will not be threatened, and they call the resulting aesthetic state *prettiness*. By contrast, *beauty* is perceived when a stimulus is novel or complex, but we anticipate that we will be able to grasp it, and we make progress toward doing so. A similar contrast was offered by Graf and Landwehr (2017), who argued that *aesthetic pleasure* is the result of fluency, whereas *aesthetic interest* reflects “the reduction of disfluency during controlled processing” (p. 2). But prettiness and interest hardly begin to exhaust the range of aesthetic responses: Where are amusement, admiration, awe in these fluency-based accounts?

Further, it is unclear how these models address specifically aesthetic experience. They seem to describe general cognitive processes that are applicable to most encounters with the world. “Identifying the physical identity of a stimulus” is something we do countless times a day without supposing it has anything to do with beauty. Armstrong and Detweiler-Bedell’s (2008) formulation implies that an easy Monday crossword puzzle is pretty, whereas a challenging Saturday puzzle is beautiful. Surely there is something that distinguishes the encounter with beauty from the smug satisfaction of a successful puzzle-solver.

The limitations of conventional experimental aesthetics are also evident in its methods. In a recent study, Christensen et al. (2020) presented images of 20 Picasso paintings for two seconds each and asked respondents to rate their beauty and creativity. Art critics, who spend decades pondering a single artwork, might legitimately wonder what respondents can make of a Picasso in two seconds. We know that participants in such studies do make meaningful judgments; the consistency of fluency effects across studies shows this, as does the distinctiveness of judgments of creativity from those of beauty that Christensen and colleagues demonstrated. But Cupchik and Winston (1996) suggested that a pleasurable response made to fluent stimuli is distinct from a true aesthetic experience, in which perceivers become actively involved in interpreting the work, discovering the relations between content and the form used to express it, and so on. They pointed out that most research in experimental aesthetics is conducted using respondents untrained in the arts, many of whom would not be able to appreciate the aesthetic value of a work even given unlimited time. As Juslin (2013) wrote, “It remains an open question whether earlier studies in the field have insufficiently distinguished between “aesthetic judgment” and (mere) preference…” (p. 255).

It is disheartening to consider the possibility that so much experimental effort has been expended on attempts to understand the wrong phenomenon, but it would not be unprecedented in psychology. For decades, psychologists conducted research on trigrams (née *nonsense syllables*) in an attempt to understand language learning, yet learned almost nothing (Bruner, 2004).

There is, however, another contemporary perspective on cognition and aesthetics. Perlovsky (2014) and Schoeller (2015) have argued that there is a fundamental knowledge instinct that normally operates at an unconscious level. In some instances, the satisfaction of the instinct reaches consciousness and is experienced as aesthetic emotion, specifically chills. These authors and their colleagues (e.g., Schoeller et al., 2024b) have focused on the neurobiology of aesthetics, which is beyond the scope of the present review.

### Affect and emotion

The fact that aesthetic experience is closely tied to affect is obvious. A tragedy may be deeply moving. Music is said to be the language of emotion (Langer, 1957). A glimpse of a Picasso may be pleasing or interesting. It has, however, proven challenging to conceptualize correctly the nature of the relationship—or relationships—between aesthetics and emotion. A number of recent accounts (Cupchik, 1994; Juslin, 2013; Tan, 2000) have made some progress. The tasks for an affective theory of aesthetics are to explain the ways in which emotion is and is not involved in aesthetic experience (Menninghaus et al., 2019); to create a taxonomy of aesthetic emotions (Fingerhut and Prinz, 2020); and to show how characteristics of the object and psychological processes in the perceiver produce specific emotions (Gabrielsson and Lindström, 2001).

Tan (2000) provided a useful convention in his terminology for emotion, distinguishing between R-emotions and A-emotions. The former are the emotions *represented* in the object: the grief depicted by a tragedy, the jollity expressed in a musical scherzo, the tranquility portrayed in a dreamy landscape. These are sometimes referred to as *perceived emotions*. There is a sense in which we feel R-emotions, by a kind of empathy or affective imagination, just as we see in our mind’s eye a house described in a novel. But R-emotions are not ours, and must be distinguished from our E- (or *everyday*; Juslin, 2013) emotions.^1^

Tan’s A-emotions are those stimulated by the *artifact*—the play, composition, or painting regarded as a work of art. (Note that both E- and A- emotions are felt, not perceived, emotions.) A-emotions include admiration, aesthetic pleasure, excitement—what Juslin (2013) and others call *aesthetic emotions*. The old paradox of why people enjoy tragedy is easily answered by saying that they are experiencing both the R-emotion of grief and the A-emotion of being moved (Hanich et al., 2014). When the A- and R-emotions are similar—when, say, we are pleased and amused by a jolly scherzo—this distinction is not obvious, but presumably still holds.

Menninghaus et al. (2019) distinguished between aesthetic emotions and art-elicited emotions—non-aesthetic responses to a work of art. Juslin (2013) pointed to several mechanisms by which music can arouse E-emotions—for example, a listener can have fond memories associated with a song from childhood. As I will argue below, many of the negative reactions to art identified by Silvia (2009), including anger, confusion, and disgust, are best regarded as art-elicited E-emotions rather than A-emotions.

### Cognition, affect, and judgment

A student taking a course in art appreciation might be taught a series of principles by which the artistic value of a work can be judged. An essay assignment to critique a painting might then be graded on the student’s discussion of symmetry and composition, accuracy of linear perspective, symbolic references, or originality. Very good essays might be written by students who were absolutely indifferent to beauty, and in whom paintings they judged to be great art evoked no aesthetic emotions at all.

But many writers argue that there is a direct connection between aesthetic emotions and judgments. Menninghaus et al. (2019) followed Kant (1790/1952) in regarding aesthetic emotions as the result and criterion of aesthetic judgment. As Fingerhut and Prinz (2020) pointed out, “One cannot call a work pleasing, interesting, or sublime without praising it” (p. 229). Aesthetic emotions are thus the subjective basis of aesthetic judgment, just as symmetry, symbolism, and originality are objective bases. In principle, the two ought to agree: Aesthetic theoreticians seek to identify objective characteristics that reliably (though not inevitably) evoke aesthetic emotions. When an individual’s objective and subjective judgments diverge, they may create what are called “guilty pleasures” (Goffin and Cova, 2019).

Leder et al. (2004) offered a model of aesthetic appreciation which focused on the challenge of understanding, and may explain the appeal of contemporary non-representational art to its aficionados. Abstract art is typically grounded in some aesthetic theory, such as cubism or conceptualism; those with expertise in such matters use their knowledge to understand the work. A satisfying interpretation is the source of pleasure, which Leder and colleagues regarded as aesthetic emotion. This is a more intellectually sophisticated version of the pleasure people take in the fluent processing of symmetric, clear, or familiar stimuli.

In a follow-up article ten years later, Leder and Nadal (2014) conceded that there is more to aesthetic emotion than intellectual satisfaction, but they did not offer an explanation of how expert judgments of art produce awe or fascination or chills. A theory of aesthetic judgment is not sufficient as a theory of aesthetic experience.

### Volition and disinterest

Gimme the beat boys to free my soul, I wanna get lost in your rock and roll and drift away.

– M. Williams, “Drift Away”

Many readers will recall the chorus of the classic rock song, “Drift Away” (Gray, 1973), in which the singer asks the band to provide the driving rhythm that will liberate him, and says he wishes to lose himself in their music to escape his troubles. That, in a nutshell, is the volitional view of aesthetic experience.

Just as *cognition* subsumes a variety of psychological processes from perception to episodic memory to logical reasoning, so *volition* refers to a range of concepts having to do with mental pulls and pushes. Motivation, desire, suspense, self-control are all volitional terms; all deal with the forces that drive psychological processes and behavior.

Following contemporary appraisal theories (e.g., Scherer and Moors, 2019), human psychological functioning can be characterized as a process in which a person with desires and aversions encounters a series of life situations. The person assesses the situation and tries to determine an optimal course of action that will maximize goal achievement and minimize adverse effects. These assessments prompt emotional reactions that may inform understanding of the situation and may motivate behavior. People are generally indifferent to situations that have no relevance to their personal agenda and simply ignore them. The life course is thus rather like completing a questionnaire, deciding with each successive item to endorse *agree, disagree*, or *not applicable.*

Something very different happens during aesthetic experience. At least since Kant (1790/1952), aesthetic experience has been characterized as *disinterested*, meaning that the object has no implications for the advancement of our agenda (Cupchik and Winston, 1996). It falls in the category of *not applicable*. Despite this, beautiful objects command our interest and attention, sometimes for hours on end. Our cognition, affect, and behavior are now driven not by our own hopes and fears, but by the dictates of the object. It is in this sense that the aesthetic experience is essentially volitional: “We give ourselves over so that… the work takes control of our responses” (Fingerhut and Prinz, 2020, p. 232).

It is volition that accounts for the difference between the vicarious R-emotions evoked by aesthetic objects and the E-emotions of daily life. One of the defining characteristics of emotions is their associated action tendencies. An actual encounter with a mass shooter would surely inspire terror and a desperate attempt to flee. But as Chatterjee (2014) pointed out, we do not run from the movie theater when such an event is portrayed. He argued that brain systems involved in evaluating situations and in propelling behavior, normally closely linked, become dissociated during aesthetic experience. It is this, rather than the intensity of the response, that distinguishes R-emotions from E-emotions (Humbert-Droz et al., 2020).

Sarasso et al. (2020) note a similar phenomenon. In aesthetic experience, motor behavior is minimized and perceptual processing is enhanced. These authors see the inhibition of behavior as a means to the end of learning: “stopping for knowledge.” From a volitional perspective, the inhibition—the suspension of the will—is the goal, and perception merely the mechanism that facilitates it.

Why would we surrender our will?—why is this altered mental state somehow desirable, such that we seek out opportunities to achieve it and treasure the art, music, and literature that creates it? For Schopenhauer (1859/1969), life is defined by the tyranny of the will; we suffer when we lack what we want, and are bored when we have it. In aesthetic experience, our own will is silenced, our ego eclipsed, and we are—at least temporarily—freed. Art is escapist in the best sense. Those with less pessimistic views of human life may still grant that aesthetic experiences are somehow refreshing; they bring rest and relaxation to our routine goal pursuit.

Contemporary psychologists might describe aesthetic escapism as a form of emotion regulation (Gross, 2015). Surely some people do listen to music or read a novel in an attempt to distract themselves from personal problems, much as others turn to drugs or alcohol. But this probably accounts for a small part of engagement with aesthetic objects, which most perceivers would attribute to the intrinsic interest (or loveliness, or humor) of the work.

In both these cases, it might be argued that seeking aesthetic experience is not disinterested, but rather an instrumental activity with the goal of inducing a painless or pleasant state. However, this differs from ordinary purposeful behavior, in which we manage our affective states by altering our circumstances to square with our personal agenda. In aesthetic experience we adopt the object’s agenda.

## Aesthetic qualities and responses: the varieties of aesthetic experience

### A taxonomy of aesthetic qualities

In an influential article, Sibley (1959) introduced the notion of *aesthetic* and *non-aesthetic concepts*. The former refers to terms that denote particular aesthetic perceptions (e.g., charming, awesome, evocative); the latter to objective features of the object (e.g., multi-colored, loud, picaresque). Sibley was chiefly interested in the philosophical status of aesthetic concepts—whether, for example, we should expect consensual judgment about them. I am more concerned with the varieties of aesthetic concepts, and how they might be organized into categories of qualities which determine the type of aesthetic response the object evokes.

Traditionally, aesthetics has been defined as the study of the beautiful. In that philosophical sense, *beauty* refers to anything that can evoke an aesthetic response, including the blinding of Oedipus, an oncoming tidal wave, or Euler’s formula. Judging from the examples they nominated (see Brielmann et al., 2021), most laypersons appear to restrict *beauty* to a particular range of attributes that might be described in such terms as *lovely, pretty*, or *picturesque*. Burke (1767/1937) contrasted the beautiful with the sublime. Battin et al. (1989) gave a longer list of distinguishable qualities, including “the elegant, the comical, the delightful, the dainty” (p. 33).

Table 1 gives a provisional list of aesthetic qualities (or virtues; Menninghaus et al., 2019), generated by listing descriptors of aesthetic objects and grouping them into categories that seemed distinctive. My intent here is not to provide a comprehensive taxonomy of aesthetic qualities, but to suggest the wide range of ways in which an object can be beautiful. An adequate psychological theory of aesthetics must account for all the varieties of aesthetic experience. A first step in understanding the categories in Table 1 would be to identify the underlying theme common to the descriptors in each.

**TABLE 1:** Categories of aesthetic qualities with descriptors (“aesthetic concepts”).

**Lovely**. Beautiful, charming, pretty, dainty, picturesque, delicate, cute, exquisite, gorgeous
**Sublime**. Majestic, grand, glorious, awesome, imposing, overpowering
**Fascinating**. Interesting, intriguing, catchy, mesmerizing, gripping, suspenseful, exciting
**Novel**. Varied, contrasting, surprising, original, ∼monotonous, ∼predictable, ∼obvious
**Elegant**. Concise, apt, neat, handsome, succinct, precise, admirable
**Mysterious**. Mystical, baffling, evocative, nostalgic, suggestive, numinous, eerie, haunting
**Moving.** Sentimental, emotional, touching, poignant, heartwarming
**Comic**. Funny, witty, amusing, hilarious, grotesque, droll, entertaining
**Harmonious**. Organic, balanced, symmetrical, rhythmic, organized, unified

#### The lovely

Burke (1767/1937) associated beauty most directly with the form and appearance of women, where it inspires love; but he extended the term to cover “all such qualities in things as induce in us a sense of affection and tenderness” (p. 45). Note that here an aesthetic quality is defined in terms of the emotion it evokes (cf. Menninghaus et al., 2019).

#### The sublime

By contrast, the sublime inspires awe rather than affection, and is, according to Burke, more closely associated with fear. The wild winds and lightning of a derecho pose real and serious threats, but they may also be psychologically electrifying. A looming mountain peak is not dangerous, but it may threaten our sense of self-importance (Arcangeli et al., 2018).

#### The fascinating

Tan’s (2000) claim that “interest is the dominant emotion in experiencing all kinds of art works” (pp. 112–123) may be excessive, but in some cases intense interest is the predominant response. Sometimes this takes the form of ongoing uncertainty or unpredictability; then fascination is a temporally-extended surprise. But the highly repetitive can also be mesmerizing.

#### The novel

Originality is prized in an artist, because it gives the aesthetic perceiver a fresh view of the world. Variety and contrast within a work are almost essential, particularly in those that require our attention for an extended time, such as a play or symphony. Meyer (1956) argued that the power of music lay in the expectations it generated, and that these were as effective when they were cleverly thwarted as when they were fulfilled.

#### The elegant

Elegance implies both perfection and economy, as in some mathematical proofs. There is some suggestion of coldness, stiffness, and formality in terms like *neat, precise*, and *apt*, and elegance seems in some sense to be the masculine counterpart to loveliness. Instead of tenderness, it evokes admiration—a kind of ego-less pride.

#### The mysterious

Mystery arises when our attention is focused on something we do not or cannot understand. A poem by T. S. Eliot may be unintelligible to us, and yet convince us that it holds a deep meaning. The mysteries of crime fiction are better labeled as “puzzles;” they present an intellectual challenge rather than evoking this aesthetic response.

#### The moving

Konečni (2008) argued that three emotional states can be induced by music: awe, thrills, and being moved. The last is characterized by an intense response that may be expressed in warmth in the chest, tightness in the throat, or tears (Zickfeld et al., 2019). Moving aesthetic objects generally depict situations that would be deeply emotional in real life—a long-delayed reunion, a bitter loss, a heroic act (Cova and Deonna, 2014).

#### The comic

Humor is itself a vast topic (see the International Society for Humor Studies),^2^ and one might argue that the funny is coordinate with the beautiful, rather than subordinate to it. However, the old pairing of comedy and tragedy as forms of drama, and the endorsement of such experts as Collingwood (1964) and Santayana (1896), who discussed at length the comic, wit, humor, and the grotesque in his treatise on beauty, justifies its inclusion as a kind of aesthetic quality. Laughter is the usual response, although laughter is also a social phenomenon probably unrelated to aesthetics.

#### The harmonious

Palmer et al. (2013) pointed out that Plato and Aristotle differed on the value of art; “[t]hey agreed, however, on the importance of unity, harmony, and integration” (p. 80). Collingwood (1964, cited in Battin et al., 1989) wrote that “the highest beauty somehow contains within itself… both the sublime and the comic, and indeed all other forms of beauty; so that these forms appear as parts of a whole” (p. 75).

A given object might have several varieties of aesthetic quality, simultaneously or successively, and the categories themselves may overlap. *Cute*, for example, seems to be a comic form of the lovely, and *surprising* may be a novel form of the fascinating.

A possible addition to the list might be The Thrilling, that is, evoking aesthetic chills (McCrae, 2007). But this would be a curious quality. Toward the end of the opening movement of the first Tchaikovsky Piano Concerto the pianist plays a rising series of fortissimo double octaves, reaching a height of E**^b^_7_** at measure 354 before hurtling downward. In Cliburn (1958) recording there is a tiny hesitation after the E**^b^_7_** that makes an exciting passage literally thrilling. But chills can also be produced by an exquisite miniature, or a poignant line of verse; they seem to result from striking and intense instances of any kind of aesthetic quality. This seems to be consistent with the views of Schoeller (2015).^3^

### Varieties of responses

Few people would respond to the question “What did you think of the concert?” with “I felt a detached involvement and sense of unity”—hallmarks of the aesthetic experience per se. Instead, the phenomenology of aesthetic experience is more concrete and more directly tied to the nature of the object. In fact, aesthetic responses are in many respects the subjective counterparts of aesthetic qualities: If a story is amusing, we respond with amusement; if a painting is charming, we will be charmed. It is therefore of interest to compare the rational classification of qualities in Table 1 with empirical data on aesthetic responses.

Schindler et al. (2017) conducted a broad and thorough review of the literature to identify aesthetic emotions and gathered data to create their aesthetic Emotions Scale.

Like Silvia (2009), who had earlier begun to examine “unusual aesthetic emotions,” Schindler and colleagues began with a very broad construal of aesthetic emotions, including negative reactions (boredom, confusion, disgust), epistemic emotions (interest, intellectual challenge, insight), and pleasing emotions (cheerfulness, amusement, relaxation) as well as what they considered prototypic aesthetic responses (awe, fascination, being moved—but not, curiously, chills). They constructed a pool of 75 items assessing 24 hypothesized aesthetic emotions, and administered it to some 500 individuals who had just experienced a play, concert, or art exhibit. A factor analysis suggested seven factors, which they interpreted as Negative Emotions, Prototypic aesthetic Emotions, Epistemic Emotions, Animation, Nostalgia/Relaxation, Sadness, and Amusement.

These all describe how respondents felt after exposure to art, but they are not necessarily what I would call *aesthetic* responses; most of them belong not in Figure 1, but in the hypothetical alternative figure describing the response of the Critic. There is a great difference between hating Iago and hating an actor’s stilted portrayal of Iago, and only the former is an aesthetic response. The Negative Emotions factor seems to represent the experience one has when an object fails to deliver an aesthetic experience (or is otherwise offensive). Epistemic Emotions, whose highest factor loading was on the item “challenged me intellectually” seems to be another non-aesthetic response to art. A challenge implies a contest, in which perceivers pit their wits against difficulties of interpretation, and feel pleased when they believe they understand the work—but that is not disinterested pleasure. Berleant (1970/2000) regarded it as “error to confuse the reflective, analytic attitude of the cognitive approach to art with the appreciative one of the experience of art” (p. 109).

The Animation factor, defined by such items as “motivated me to act” and “spurred me on,” reflects a response that some artists surely hope for—one thinks of Delacroix’s *Liberty Leading the People*. But a motivated person is one who has adopted a new goal, or strengthened an old one, and goal-directed behavior is not aesthetic. Of course, aesthetic means may be used to make an appeal more persuasive, just as songs are used to teach children lessons. Conceivably, perceivers could admire the artistic accomplishment of persuasiveness even if they are not persuaded: A socialist might enjoy an Ayn Rand novel—but the response here would be admiration, not animation.

Of the four remaining factors, three refer to moods induced in the perceiver: Nostalgia/Relaxation, Sadness, and Amusement. The first two might be seen as instances of being moved (“moved me” is a definer of Sadness), although it is not clear whether these induced moods are themselves responses, or merely byproducts of being moved. Being calm or sad is not in itself an aesthetic state.

Amusement (“was funny to me”) would seem to be correlative to the comic. The fact that it forms an entirely distinct factor from Prototypic aesthetic Emotions raises the suspicion that the comic is not, in fact, an aesthetic quality. Some forms of humor may have aesthetic aspects—wit may be elegant, a punchline may be surprising—but other forms (slapstick, perhaps) may be purely comic.

The Prototypic aesthetic Emotions factor includes a wide range of responses that correspond rather well to the proposed list of aesthetic qualities, as Table 2 shows. But the categories of aesthetic qualities are supposed to be qualitatively distinct; why, then, do they define a single factor? Recall that respondents were asked to describe their emotional reactions to an entire event (film, concert, exhibit), and it seems likely that they endorsed all items that they ever experienced. One work in an exhibit may have been lovely, another mysterious, and a third moving; an aesthetically sensitive respondent would have endorsed all three, contributing to their covariance. A well-written play might be at times elegant, at times baffling, but ultimately harmonious, whereas a poor play would be none of these. Across respondents and events, what emerged was a general aesthetic factor.

**TABLE 2:** Categories of aesthetic qualities (from Table 1), Prototypic aesthetic Emotions (PAE) items (from Schindler et al., 2017), and genre-descriptive adjectives (from Knoop et al., 2016).

Quality	PAE Items	Adjectives
Lovely	“I found it beautiful,” “Was attracted”	Beautiful, poetic, melodious
Sublime	“Was overwhelmed,” “I found it sublime”	Thrilling
Fascinating	“Fascinated me,” “Gripped me”	Dramatic, suspenseful, interesting, [not] boring, exciting, riveting
Novel	“Surprised me,” “Astonished me”	
Elegant	“I found it perfect,” “Impressed me”	Succinct, short, rhythmic
Mysterious	“Baffled me”	
Moving	“Felt deeply moved,” “Touched me”	Tragic, sad, romantic,
Comic		Witty, ironic, humorous, funny, entertaining
Harmonious	“I found it harmonious”	Harmonious

### Aesthetic qualities in the object

Qualities may be defined in terms of the responses they produce in the perceiver, but they might also be defined by features of the object. By *features*, I mean the constituent parts and their objective characteristics; they correspond to Sibley (1959) non-aesthetic concepts. O’Keeffe’s *Summer Days* depicts a deer skull and wildflowers surreally superimposed on a desert sky; the colors, except for the flowers, are generally muted; the painted surface is smooth. These features were chosen by the artist to tell a particular story, but they also determine the tone of the work, its mysterious aesthetic quality.

Burke (1767/1937) devoted a great deal of attention to the features that contributed to the perception of beauty. He rejected the idea that visual beauty was caused by proportion or perfection, and instead concluded that we see beauty in the small, smooth, delicate, and brightly (but not garishly) colored. He suggested corresponding characteristics for sound. *Lovely* music would be soft, smooth in articulation and melodic line, consonant in harmony, and moderately slow, like the Entr’acte from *Carmen* or “Morning Mood” from *Peer Gynt*.

It is possible to identify features associated with other quality categories. The musically sublime (think of Bach’s *Passacaglia and Fugue* or Strauss’s *Also Sprach Zarathustra*) is typically loud, deep in pitch, and solemn in tempo. The mysterious can be evoked by what is unsaid: According to Mason (1946), the “astounding” Moderato section of the second movement of Tchaikovsky’s *Souvenir de Florence* abjures “melody, harmony, figuration and even rhythm” and derives its haunting effect “solely from shades of monochrome and from dynamics” (p. 112).

Many aestheticians would disagree with Burke’s narrow interpretation of the features that define beauty (or the Lovely), and much of the literature on art consists of debates on what features are necessary or sufficient to define a quality. The seems to be general agreement, however, on the association of genres with qualities (Tan, 2000). Tragedies should be moving, horror stories should be scary, epigrams elegant. Knoop et al. (2016) asked German students to list adjectives that described “the aesthetics of literature,” or of a more specific genre (novels, short stories, poems, plays, comedies). A wide variety of terms were generated that distinguished the different genres: Poems were *beautiful*, novels *suspenseful*, comedies *funny*. The most frequently mentioned terms are shown in the third column of Table 2, sorted by qualities.

Aesthetic emotions do not exhaust the ways in which we respond to beauty. Art can absorb our attention (Kuijpers et al., 2017), transporting our thoughts to a new world, generating mental imagery. It can stimulate reflection and enhance our understanding of ourselves and human relationships (Oatley, 1995). It can evoke personal memories and inspire dreams.

But all this happens only if the object succeeds in creating an aesthetic experience. Many attempts to create effective art fail, even when they follow accepted formulas and incorporate relevant features. And even effective artworks can only be appreciated by perceivers who are adequately prepared and personally disposed to do so.

## Characteristics of the aesthetic experience

Although some psychologists view aesthetic experience as in principle indistinguishable from ordinary experience (e.g., Skov and Nadal, 2020; see Cupchik and Winston, 1996, for a discussion), most philosophers and many psychologists claim aesthetic experience is qualitatively different from normal experience—it is an altered state of consciousness. A number of criteria have been proposed that characterize this state, including disinterested engagement, a sense of unity and completeness, pleasure, and self-transcendence.

### Disinterested engagement

Bullough (1912) proposed that *psychical distance* is the central feature in the perception of beauty, and Palmer et al. (2013) “take aesthetics to be the study of those mental processes that underlie *disinterested* evaluative experiences” (p. 79, italics added). In ordinary experience we view the world instrumentally, as an opportunity to satisfy our needs or as an obstacle to our plans. We normally take an interest in things just to the extent that they are relevant to our goals and desires. In aesthetic experience, by contrast, objects are intrinsically interesting and may have no bearing on our personal agenda: “What’s Hecuba to him, or he to Hecuba, that he should weep for her?” asks Hamlet. It is this detachment that allows us to appreciate the derecho in spite of danger, or to relish the pathos of Barber’s *Adagio for Strings*.

A few writers have dissented from this view. Santayana (1896) rejected the idea that the appreciation of beauty is disinterested, because we have a keen interest in enjoying it—why else would we travel to museums or buy tickets to a concert? Berleant (1970/2000) recognized the intrinsic interest of art, but objected to such terms as *psychical distance*, because they “tend to place the experiencer in a distant seat where he becomes more than disinterested; he is left remote, detached, and uninvolved” (p. 123). True aesthetic experience, Berleant argued, demands total immersion in the object.

These objections do not undermine the contention that aesthetic experience is a special state marked by detached involvement. Surely there is a kind of aesthetic pleasure, but it stands apart from the normal experience of pleasure, which reflects some advancement in our goals. We are pleased when we win the lottery because we can use the money; we are pleased when we see a watercolor just because it is pretty.

*Psychical distance* has been interpreted differently by different authors. Kuijpers et al. (2017) used the term *aesthetic distance* to explain the difference between absorption in a fictional world (as when we are engrossed in a story) and attention to the writing itself, created by such literary techniques as foregrounding, estrangement, and deviation. This latter focus they consider distant, in contrast to the close absorption in the story. A similar view had been offered by Oatley (1995), who held that immersion in the story was not a true aesthetic experience, but mere entertainment. Berleant (1970/2000) would surely object that Oatley’s view seems to favor the detached aesthetic judgment of the critic over the total absorption of the engrossed reader.

Kuijpers et al. (2017) appear to be focused on attentional distance, where riveted attention is *close* and detached contemplation is *distant*. I believe Bullough’s conception of psychical distance is fundamentally volitional. The aesthetic experience occurs when we devote cognitive and emotional resources to an object that is of no practical concern to us. The *distance* is that between our personal interests and the intrinsically interesting object. Aesthetic experiences fail when the topic “cuts too close to home;” jokes are unfunny when made “too soon” after a traumatic event. Schopenhauer (1859/1969) deplored the depiction of bread or wine in still lifes because it may stimulate our appetites.

If the aesthetic state is defined by disinterested engagement, it must, like distance, be a unipolar concept. An aesthetic experience may be fleeting, intermittent, or sustained, and our engagement may be mild or intense, but it cannot be negative. An anti-aesthetic experience would be one in which we are especially concerned with our personal agenda, and that has nothing to do with aesthetics—it is ordinary experience in a high-stakes situation. Ugly art, boring stories, or off-key singing may be painful or annoying, but these are art-elicited emotions, not aesthetic emotions.

### Unity

Beardsley (1969) claimed “a person is having an aesthetic experience during a particular stretch of time if and only if the greater part of his mental activity… is united and made pleasurable by being tied to the form and qualities” (p. 6) of the object. The key term in this definition is *united*. Beardsley believed that an aesthetic experience must have “coherence and completeness” (p. 6); like the ancient Greeks, he saw beauty in the organic and harmonious unity of the object, which produces a corresponding unity of experience in the perceiver. This unity is enhanced by detachment from our routine life, where multi-tasking is perhaps the norm.

It may be that coherence and completeness leads to the highest form of aesthetic experience, the most intense and satisfying. But in my view, harmoniousness is only one category of quality, not the *sine qua non* of all aesthetic experience. The Romantics, in fact, were particularly fond of the incomplete and fragmentary (Rosen, 1995)—think of Coleridge’s “Xanadu”—and the *Belvedere Torso* has been admired for centuries.

An aesthetic experience, then, need not be complete, intense, or extended in time. The flash of a goldfish in a pond may ignite a brief flash of aesthetic experience. To sensitive individuals, something as mundane as patches on broken panes of glass can trigger an aesthetic experience (Ginsberg, 1986). Such transient and incidental aesthetic experiences—skillfully arranged—can become the ingredients of a larger experience: A poet may tell a suspenseful story, using vivid metaphors set in rhyming iambic pentameter. This was the view of Osborne (1977, quoted in Mitias, 1986), who argued that the total effect of an artwork is usually created by many subsidiary effects: “Most works of art are complex constructs with aesthetic qualities at various levels…. The work of art… has overall aesthetic qualities and the contained parts have also their aesthetic qualities” (p. 245). If these contained parts mutually reinforce one another, the work will have a kind of unity that is one more source of aesthetic pleasure.

### Pleasure

In addition to being “united,” Beardsley (1969) also characterized the aesthetic experience as “pleasurable,” a feature emphasized by Santayana (1896). Some art may be edifying or inspiring, but in general we read stories or visit museums because we enjoy it. The question is whether this kind of pleasure is qualitatively different from the pleasure we take in eating ice cream or winning an election. There are two reasons to think that it is: It is not utilitarian, and it can be provoked by situations that seemingly should be distressing: sad music, tragic stories, scary movies (Clasen et al., 2020).

Pleasure (or more broadly, positive valence; Russell, 1980), is usually the affective aspect of the appraisal that we are nearing or have reached the satisfaction of our needs or goals (Carver, 2015), which may be biological, personal, or moral. We act as we do with the expectation that it will bring us closer to reaching these goals, and in that sense our usual acts are all more or less utilitarian. But unless one posits an intrinsic need for aesthetic experience, the contemplation of art does not normally further our personal aims—yet it commands our attention in ways that show it is important to us.

### Self-transcendence

If psychical distance is to be distinguished from indifference, it suggests that the focus of experience has merely shifted from one’s personal concerns to an interest in the object in itself. This is implied in Beardsley’s (1969) claim that when a person has an aesthetic experience, “the greater part of his mental activity… [is] tied to the form and qualities” of the object. There are two consequences: First, the quality of the experience is distinguished and heightened: As Canaday (1980) wrote, the function of art is “to clarify, intensify, or otherwise enlarge our experience” (p. 5).

The second consequence is that as we are absorbed in the object, we step outside ourselves. This is a characteristic about which writers wax poetic. Ginsberg (1986) claimed that “aesthetic discovery is liberation from the ordinary bonds of the earth. It is elevation and alleviation of the spirit” (p. 64). Osborne (1986) noted that intense aesthetic experiences occur when “a lapse of personal identity is combined with a feeling of metaphysical oneness with the universe” (p. 129). All of this is reminiscent of Schopenhauer’s (1859/1969) view that aesthetic contemplation is virtually the only way to escape—at least temporarily—the tyranny of the will, and its attendant cycle of suffering and ennui.

### Temporal course

At length the Vision closes; and the mind, Not undisturbed by the delight it feels, Which slowly settles into peaceful calm, Is left to muse upon the solemn scene.

— W. Wordsworth, “A Night-piece”

To say that an aesthetic experience is pleasant, or unified, or transcendent seems to suggest that it is a single and uniform state. Like any experience, however, it must occur over time, and there is every reason to think that the nature of the experience changes—evolves, vacillates, or dissipates. Ingarden (1961) in particular argued that there is a regular (and quite complicated) sequence of reactions by the perceiver. On encountering a visual artwork, we may be struck by some feature (color, contour, representation) which arouses in us interest or excitement. This leads to a focus of our attention on the object and a concomitant isolation from our mundane concerns. Attention to the object means that we study it in depth and attempt to grasp how its component elements cohere into an organic unity. When we detect such unity we experience intense pleasure. The final phase of the experience, according to Ingarden, (and Wordsworth) is “a rather quiet *gazing upon* (contemplating) the qualitative harmony” (p. 308) of the object, and a sense of admiration and satisfaction.

Ingarden’s basic premise—that the experience changes over time—is surely correct, but it seems unlikely that there is a fixed set or sequence of responses. Sometimes we study a picture not because something in it strikes us, but because we have heard that this is art worthy of our attention. In the performing arts, the perceiver submits passively to the drama or music as it unfolds, and the experience of hearing a piece of music for the third time is far different from hearing it for the first time.

## Aesthetic devices and psychological mechanisms

### Features and devices

Artists (Forster, 1927), critics (Canaday, 1980), and psychologists (McManus, 2005) have devoted enormous attention to the formal and technical means by which artists elicit aesthetic responses. Painters use symmetry, chiaroscuro, and linear perspective; composers use rhythm, dynamic contrast, and melodic imitation; poets use rhyme, symbolism, and figures of speech (of which Wikipedia lists 200+). A complete catalog of such devices would be encyclopedic in length. Learning about these devices is a major focus of training in the arts and art appreciation.

Devices are distinct from aesthetic features. As shown in Figure 1, *devices* and *features* are both components of the aesthetic object. Features determine the quality of the object and thus the nature of the aesthetic response. Pastel hues might help make a painting *lovely* rather than *mysterious* or *sublime*. Features also contribute to the generation of R-emotions: Quick tempos and bright timbres convey cheerfulness.

In contrast, *devices* operate through psychological mechanisms to induce the aesthetic state—a will-less, disinterested absorption in the object. Neither slow nor quick tempos can themselves create such an effect, but rhythm—the regular, repetitive, hypnotic pulse of music—can.^4^ Try listening to a march and tapping your toes to a different beat. And note the selflessness of the toe-tapping response itself: Whoever would include it in their personal agenda? Rhythm is an aesthetic device.

### Investigating psychological mechanisms

Schopenhauer (1859/1969) offered an account of how the aesthetic state is produced, but it is metaphysical rather than psychological. Through the genius of the artist an object is created that facilitates the perception of a Platonic Idea. For the receptive viewer, Cezanne’s apples are not relatively crude reproductions of an assembly of fruits; they are images that express the eternal essence of The Apple. So compelling is this vision that the perceiver becomes a “pure, will-less, timeless subject of knowing” (p. 199). Odd as this explanation may seem to psychologists, it has certain similarities to psychological accounts. Platonic Ideas are the philosophical prototypes of material objects, and prototypicality is empirically associated with beauty (Martindale and Moore, 1988). The pleasure we experience from contemplating Ideas is akin to that derived from fluent processing of an image (Reber et al., 2004). Still, a psychological account will differ in many respects.

Figure 1 proposes that devices operate through psychological mechanisms, and a theory of aesthetic experience requires some account of them. Several conceptual models of encounters with art have recently been proposed (Pelowski et al., 2016). Most of them are essentially cognitive and describe stages by which the object is perceived, interpreted, and evaluated. Presumably because they were developed by psychologists, these theories have focused almost exclusively on the processes that occur in the perceiver, rather than on characteristics of the object. A model offered by Pelowski et al. (2017) is intended to account for interactions with visual art in general and makes no differentiation between sculpture and painting, or between religious iconography and Pop Art. There may be a sense in which responding to such different art forms is uniform, but so global a model is unlikely to be very informative. Surely the mechanisms by which a clever joke induces aesthetic experience are different from those operating in the appreciation of a Beethoven symphony.

Psychologists do attend to some aspects of the stimulus—studies of fluency involved manipulation of such variables as symmetry, familiarity, and complexity. But these form a very restricted subset of aesthetic devices. Students of the humanities—scholars in such fields as art criticism, literary theory, and film studies—focus their attention on aspects of artworks that produce distinctive effects in perceivers. Tan (2000) has proposed that psychologists collaborate with these scholars to understand the ways artworks elicit A-emotions: “the social scientist’s work is to reconstruct the emotional process from the characteristics of the stimulus that have been analyzed by the humanist” (p. 117).

Perhaps the best example of this approach comes from the work of literary scholars in the tradition of cognitive poetics (Harrison and Stockwell, 2014; Tsur, 2008). Cognitive poetics is an attempt to understand how features of the text affect readers in general, and how individual readers respond uniquely to texts. It differs from traditional literary criticism by drawing explicitly on cognitive psychology and linguistics, and is thus amenable to empirical tests (e.g., Obermeier et al., 2013). Although there are exceptions (e.g., Oatley, 2003), most of its practitioners appear to be specialists in literature rather than in psychology, and the vocabulary they use (*resonance, deictic shift, suggestion structure, readerliness*; see Harrison and Stockwell, 2014) is likely to be unfamiliar to most cognitive psychologists—a potential obstacle to collaboration.

Once the scope of aesthetic devices has been properly expanded, empirical investigation requires theoretical guidance. How many studies (with replications!) would be needed to determine the mechanisms that account for the power of each of the 200+ figures of speech? From the volitional conception of aesthetic experience offered here, it is possible to theorize about how specific devices operate, and from these proposed accounts testable hypotheses might be derived. If a range of devices from different media were studied, the heuristic value of the volitional approach itself could be assessed. Here I will offer suggestions about how a few important devices work.

### Possible mechanisms

Quite a few… poetic effects are the result of some drastic interference with, or at least delay of, the regular course of cognitive processes.

— Tsur (2002, p. 281)

#### Rhyme

Consider rhyme, a poetic device in which syllables, generally at the end of a fixed-meter line, sound alike. What is the attraction of this device? First, it sets the poem apart from prose by calling attention to acoustic features of language. The sounds in normal speech are transparent; we hear through them to focus purely on the sense. Rhyme’s reminder that words have aural as well as semantic features is a surprise, and momentarily arrests our perception. It also slows our information processing, because we must now attend to two aspects of language. These effects are of course shared by other sound-related devices, such as rhythm and alliteration.

When used in conjunction with regular meter, rhyme also engages a set of expectations, which Meyer (1956) has shown are central to our appreciation of music. In poetry, we anticipate the return of a particular sound, perhaps unconsciously considering candidates that would suit both sound and sense. We are gratified when our expectations are fulfilled, and momentarily taken aback when they are delayed (as in ABAB rhyme schemes) or denied (as in censored rhymes).

Very young children can grasp rhymed couplets, perhaps as a kind of common fate Gestalt. Expectations generated by more elaborate rhyme schemes, such as Dante’s terza rima or Pushkin’s Onegin stanza, clearly depend on a knowledge of poetic conventions. Although having an aesthetic experience is quite different from constructing a critical analysis of an object, it usually presupposes considerable training and experience (Osborne, 1986). The basic psychological effects of expectations fulfilled or denied are always the same, but the expectations themselves may depend on a properly prepared mind.

#### Imitation

Probably the most fundamental device in visual art is imitation, the recognizable representation of persons, places, or things. Subject matter, style, and skill, of course, are crucial to the evaluation and enjoyment of an object, but more basic is the mere fact that we perceive an image as both like and unlike some real object (or hypothetically real object, such as Pegasus). Drawings and paintings, in particular, differ from the objects they portray chiefly because they are two-dimensional and static, and it is as essential that we notice these properties as it is that we recognize the object portrayed. A trompe l’oeil painting may be quite unremarkable until we realize it is a mere image.

In normal perception we instantly recognize what we see and almost as quickly appraise its relevance and importance: A familiar tree in the yard is ignored; a friend spotted in a crowd may prompt a smile; a slithering snake evokes Dickenson’s “tighter Breathing / And Zero at the Bone.” Surely these appraisals and responses are also set off by a picture of a tree, a portrait of a friend, a sketch of a snake—and as surely, they are inhibited by the realization that these are only images. We have been tricked and our usual psychological routine disrupted.

One effect of this disruption is that it calls attention to the object. Once I notice that this is only a picture of my friend, I need not think about what to say or whether to wave; the perception ceases to be a cue and becomes a focus of interest in itself. Whether and how much that interest is sustained depends on other aesthetic features of the image—its symmetry, or stylization, or color composition.

Flooded as we are today by images from phones, films, and magazines, the aesthetic power of imitation must be nearly exhausted. But imagine the effects on early visitors to the caves at Lascaux: They must have been dazzled and awed by these beasts-that-are-not-beasts. What is a cheap trick for us must have been a profound mystery for them.

It might be argued that the true device of imitation is not mere representation, but the artful way in which the image captures its subject. This might be seen in the photorealism of Van Eyck or Holbein or the insightful caricatures of Hirschfeld. What we experience here is a form of the elegant: We are amazed and pleased by the demonstration of skill, even though it is of no use to us.

#### Repetition

Novelty and repetition are the yin and yang of aesthetics. The lure of novelty is fairly clear: We are surprised, struck, intrigued by something new; this is the beginning of aesthetic experience, according to Ingarden (1961). Repetition charms us, in part, by satisfying our expectations, as noted in the case of rhyme. It is hardly surprising that sophisticated art often includes variations, in which an idea or musical theme is repeated with a different twist: it is then both familiar and novel.

But there must be something more than expectation that makes repetition compelling. We can anticipate the punchline of an old joke, but feel no thrill when we hear it. Reruns of most television programs are lackluster. But children, and many adults, will happily sing the quintessentially repetitious “Row, row, row your boat” over and over. Expectation is a volitional affect (McCrae, 2021); we feel a certain tension waiting for release by the expected event. But repetition has a more relaxed appeal. Because we know what is coming, the work of perceiving and interpreting what we hear or see is reduced; we are carried along like a train on its tracks. It is this passivity that makes repetition an effective device.

What is it that distinguishes the welcome familiar from the tedious and stale? At least in part, it is the other aesthetic qualities of the piece. Simple though it is, “Row” is a well-crafted melody, with its ascending and descending halves; and sung as a round it has the extra attraction of the dizziness of our divided attention. Even witticisms bear repeating if they are clever enough—Twain’s definition of life as “one damned thing after another” is still funny. Could we identify with certainty the features that make repetition attractive we would have the elusive formula for the hook.

Clearly, there is a limit to the aesthetic power of repetition: Musical classics become warhorses, and inspired metaphors become clichés. Curiously, the more powerful the initial impression, the quicker it seems to fade: Romantic music is less durable than Baroque. One of the constant challenges for performers is to find ways to make familiar material fresh, through new interpretations or adaptations. There is an art to managing devices.

#### Narrative

Narrative is to literature something as imitation is to the visual arts. It is the representation of persons (or, more broadly, characters) interacting over time with others and the world. The mechanisms relevant to imitation—the disconcerting experience of illusion and the admiration of a skillful depiction—are also found in narrative, particularly in the delineation of character, but the fact that narratives occur over time adds other complexities.

Forster (1927) distinguished between “story” and “plot.” The story is a mere chronology of events, which sustains interest solely by our curiosity about what happened next. A plot, in his terminology, is a story in which the focus of interest is on *why* events occurred—a focus that engages the perceiver’s memory and intelligence.

Forster regarded the story as “the lowest and simplest of literary organisms” (pp. 27–28) and implied that the mere satisfaction of curiosity does not rise to the level of the aesthetic. This seems an oversimplification. In normal life, curiosity is functional: We read the weather report to learn what to wear, and the newspaper to keep abreast of the state of the world we live in. But why would we be curious about the fate of Little Red Riding Hood?

The usual explanation is that we identify with her, at least in the very basic sense that we are both human beings, and like her we face unknown dangers. Note that this is a kind of out-of-body experience: We set aside for the moment our own identity and interests and take on the avatar of a little girl. This is a remarkable phenomenon. We do not normally identify in this way with strangers on the street or even with our intimate associates. It is true that there are features that facilitate identification: We say that some characters are sympathetic because they resemble us, or have traits we aspire to; but this could also be said of many people we know but do not identify with.

It seems likely that we must have learned at an early age that there are rewards for adopting this highly artificial attitude. If we are promised a story, we give our attention and take an interest in the characters and their experiences—a sort of willing adoption of belief. Provided the story sustains our interest by suspense, surprising turns of events, or moving responses in characters about whom we care, we find ourselves absorbed in the narrative and correspondingly liberated from our own personal concerns.

Narrative is the overarching device of fiction, but it must be supported, or decorated, by a host of other devices, including suspense, evocation of emotion, deftness of characterization, organization of the story line, and beauty of language. Different writers and different genres emphasize different subsidiary devices, and different readers prefer them.

From this perspective, what Forster (1927) called “plot” is only another embellishment of narrative. In a novel with a well-constructed plot (Forster cites Meredith’s *The Egoist* as an example), each event and each detail has some significance, but what this is may not become clear until much later, when its role in the plot is revealed. Murder mysteries are a prime example of novels with plots, although there the focus is on *who*, not *why*. The essential point is that the reader is invited to be attentive to details, to ponder possible outcomes, to wonder about odd or unexpected events.

As in music, there are aesthetic rewards associated with the expected (or unexpected) denouement; in fiction, more than in music, there is also the retrospective appreciation of the foreshadowing: “Ah, I see, now it all makes sense.” But Forster is chiefly concerned with a subtler device. The attention demanded of the reader carries with it a kind of confusion: “To appreciate a mystery, part of the mind must be left behind, brooding, while the other part goes marching on” (p. 87). In real life this state of mind would not generally be pleasant. The scientist faced with discrepant results continues to ponder them while gathering new data, but the experience is likely to be discouraging or frustrating, rather than interesting. It is only because readers have no real stake in the outcome that they can enjoy being bemused.

#### Symmetry

Symmetry is a fundamental device for the visual arts, and has echoes in literature and music—for example, the ABA form of many songs. There are many different kinds of symmetry, including the sheer repetition of a honeycomb, the radial symmetry of a starfish, and the mirror-image symmetry of a face. In aesthetics, the term is sometimes used more broadly to include balance in composition, centeredness in a frame, and so on. The essence of symmetry is that it embodies a pattern that simplifies processing of the image: Once we know how the left side of a face looks, we can immediately envision the right.

Symmetry has been extensively studied in experimental aesthetics and we know a good deal about how it affects viewers. Ognjenović (1991) showed that viewers perceived and appreciated symmetry in images shown for a few milliseconds; they required longer exposure to appreciate elaborateness of design. Leder et al. (2019) presented laypersons and artists with both symmetric and asymmetric images; the former preferred symmetric, the latter, asymmetric. This is the familiar contest of familiarity versus novelty, and suggests that symmetry is a simple device appealing chiefly to naïve perceivers. Its effect is quick but transient.

Human perceptual systems are designed to seek and recognize patterns, so symmetry functions as a device in part by attracting attention and arousing interest in the object. In a successful painting, other devices (imitation, implied narrative, symbolism, etc.) must sustain interest. But symmetry is also a source of the sense of unity: Interesting visual details form part of a single experience because they are bound together in compositional balance. It seems likely that many viewers alternate between examining particular features of a work and stepping back to see the “big picture” created by symmetry.

### The general principle

*The Outer Limits*, a science fiction series of the 1960s, began each episode with the claim that they would commandeer our television set: “We will control the horizontal. We will control the vertical…. For the next hour, sit quietly and we will control all that you see and hear”.^5^ This is precisely the logic of aesthetic devices: They manipulate our attention, memory, information processing, appraisal, and affect, sometimes producing a state Barthes (1975) called *drifting*, which occurs “whenever, by dint of seeming driven about by language’s illusions, seductions, and intimidations, like a cork on the waves, I remain motionless” (p. 18). The effect is to shift the focus from our personal agenda to the aesthetic object and its agenda. We are taken out of ourselves and our ceaseless “getting and spending,” given a vicarious new life, presented with something so remarkable that we admire it without envy.

Most devices can be regarded as tricks, and in themselves have limited interest. Yes, our attention is held while the dominant seventh chord resolves into the tonic, but few people would describe this as an instance of musical beauty. But great artists, drawing upon forms and techniques developed over centuries, can construct from these simple elements large organic structures that have powerful and memorable effects.

## Individual differences

The difference between most people and myself is that for me the “dividing walls” are transparent.

— Jung (1961, p. 355).

The discussion of mechanisms assumed a single human psychology: Laws of Gestalt, processes of appraisal, volitional affects were considered to be the same for everyone. In fact, of course, that is never true, and famously so for art appreciation, where *de gustibus non disputandum est*. People differ markedly in their preferences for different art forms, for different styles in those forms, for different artists working in those styles.

These differences are in large measure learned. Exposure to songs, stories, and pictures is an important part of children’s enculturation, and most people prefer the styles of their own culture: Few Americans fully appreciate Indian ragas, still less the musical accompaniment to Noh plays^6^ Many aesthetic devices depend on learned conventions, such as the symbolism of flowers or the repeat structure of minuets, and classes in art or music appreciation are intended in part to teach these conventions. Lifelong encounters with objects in libraries, museums, and concert halls, perhaps with commentary by curators and reviewers, refine aesthetic tastes.

But some individual differences in aesthetic experience have an innate basis. Rentfrow and Gosling (2003) have shown that musical preferences are associated with heritable personality and cognitive traits: Agreeable people like upbeat and conventional music; extraverts enjoy energetic and rhythmic music. But the most important determinant of how people respond to art is another personality dimension: Openness to Experience, which “sits at the center of the psychology of aesthetics, creativity, and the arts” (Silvia et al., 2015, p. 377).

Openness is a broad collection of normally-distributed traits characterized by an exploratory interest in various aspects of the world. Open people have a vivid imagination, deep and complex emotions, a preference for variety, intellectual curiosity, and unconventional values. McCrae and Costa (2010) measure includes a subscale of Openness to aesthetics, assessed by items concerning appreciation of, and characteristic responses to, art and nature. One item asks about experiencing “a chill or wave of excitement” in response to poetry or art; it is the single most diagnostic of 48 Openness items in a wide variety of cultures (McCrae, 2007).

McCrae and Costa (1997) argued that Openness has two aspects: motivational and structural. The motivational aspect explains the attraction of aesthetic experience to Open people. They have a strong need for variety, an interest in encountering and developing new ideas, a fascination with examining details. It is thus little wonder that they gravitate to the arts, where originality is at a premium, where literature invites them to explore new worlds, where the complexities and subtleties of music are endlessly intriguing.

Closed people have very different motives. They want clear and simple answers, and often seize on the first available (Kruglanski and Webster, 1996). Tsur (2002) noted that “Persons who are intolerant of uncertainty or ambiguity may seek rapid [conceptual] categorisation and miss some of the most crucial aesthetic qualities in poetry” (p. 280). Two seconds may be quite sufficient for them to form an opinion on a Picasso painting.

The second, structural aspect of Openness is less easy to describe. Rokeach (1960) argued that dogmatic (i.e., closed) people have compartmentalized thinking, making their beliefs impervious to disconfirming information. Hartmann (1991) proposed a similar concept of mental boundaries; those with strong boundaries prefer a world with strictly separated entities: Men and women should have distinct roles; pictures should have strong outlines; thoughts and feelings should be kept separate. McCrae (1994) showed that Hartmann’s Boundary Questionnaire was strongly (*r* = 0.66) correlated with Openness to Experience, implying that open individuals have weak and permeable mental boundaries. That conclusion is consistent with evidence that Openness is associated with tolerance of ambiguity, perceptual synesthesia, and divergent thinking (McCrae, 1987; McCrae and Costa, 1997).

Do these transparent “dividing walls” facilitate the operation of the psychological mechanisms that generate aesthetic experience? Clearly, they play a role in the central features of narrative. The ability to take the perspective of characters and to identify emotionally with them requires a fluid sense of identity. Closed individuals with solid ego boundaries might identify with a character they admired (“that’s just what I would have done!”), but the scope of their literary interest would be quite limited.

It is easy to hypothesize effects of structural Openness on response to other devices. The bittersweet experiences that we find moving require the ability to feel conflicting emotions simultaneously. An appreciation of rhyme presupposes a coordinated division of attention between the aural and the semantic. The sense of unity that symmetry gives depends on the ability to toggle between specific details and the integrated whole. Mental fluidity is implicated in all these processes.

It is a useful exercise to try to imagine how a very closed individual would respond to aesthetic devices. Imitation, for example, creates the illusion that a mere image is a real object; to appreciate it, we must keep both interpretations in mind. For a closed viewer who sees the world as either black or white, that is difficult. There are stories of cinematically-naïve cowboys shooting the villain in a film because they mistook the action to be real; closed people might lose interest in a basket of fruit when they realized the apples were only wax. Clearly, it would be necessary to verify such hypotheses empirically.

## Discussion

The psychological study of aesthetics has a long history of cognitive theories and experimental methods. More recently an emphasis has been placed on affective theories of responses to art, using a broader range of research methods (Juslin, 2013). In this essay I have espoused a volitional account of aesthetic experience, arguing that familiar artistic devices such as rhyme and symmetry interrupt the normal pursuit of our personal agenda, with predictable consequences: Our attention is absorbed in the object, we mentally simulate characters’ actions and reactions, we experience emotions vicariously. In doing this we feel a variety of pleasurable aesthetic emotions and—at least sometimes—a sense of liberation and self-transcendence.

### Qualifications, limitations, and positionality

The empirical studies cited in this article were conducted almost exclusively by Western psychologists on research participants from North America and Europe, and one might conclude that the results are generalizable only to those populations. However, it is a truism of anthropology that art, music, and story-telling are pan-cultural (Brown, 1991). There is also cross-cultural evidence from a wide range of nations that the personality trait of Openness to aesthetics is found universally as part of the broader Openness to Experience factor (McCrae et al., 2005), and that aesthetic chills are found everywhere as a response to art and beauty (McCrae, 2007). These facts increase the likelihood that other aspects of aesthetic psychology may also be universal.

The philosophers on whose work I have drawn are from the Western tradition, although there are well-developed alternatives (e.g., Indian; Sen, 1976). Certainly, there are important cultural differences in the philosophy of art. Japanese Wabi-Sabi, for example, focuses on the impermanence of life by creating imperfect artworks (Koren, 1994), whereas classical Greek art strove for perfection. Such philosophical differences can be expected to influence the forms of art produced, but whether they lead to fundamental differences in psychological responses to art and beauty is unclear.

My interest in this topic was stimulated by a reading of Schopenhauer as an adolescent, which ultimately led to an undergraduate degree in philosophy. My graduate degree and subsequent career were in psychology—but not experimental psychology. These circumstances may account for my willingness to offer armchair arguments on the operation of aesthetic devices and my critical interpretation of some experimental research on aesthetic responses. My views have also been shaped by a lifelong concern for the arts, especially music.^7^ My taste, however, is traditional, and I may have failed to deal adequately with responses to contemporary art.

### Future directions

The long tradition of experimental aesthetics could be extended by a more comprehensive definition of its scope. The great majority of studies have asked respondents to judge how *pleasing* or *beautiful* a set of stimuli were; I doubt any have asked how *apt, mesmerizing*, or *haunting* they were. Would research on such alternative aesthetic qualities lead to the identification of mechanisms beyond processing fluency that are important in understanding aesthetic experience?

There is also a solid body of research on some volitional processes, especially impulse control (e.g., Mischel and Ebbesen, 1970), but there are few paradigms for research on volitional processes in aesthetic experience. One is suggested by Schopenhauer’s claim that bread in a still life would distract a hungry viewer from appreciating the sheer beauty of the work. Hunger is an easily manipulated variable; does it in fact affect aesthetic judgments? Would extrinsic rewards (such as generous compensation) decrease intrinsic appreciation of aesthetic objects? Of course, research on volitional processes need not be strictly experimental; self-reports, behavioral observations, or structured interviews may be better suited, especially for temporally extended artforms like novels or operas.

Research in the tradition of cognitive poetics (e.g., Obermeier et al., 2013) has examined some proposed mechanisms that may explain the effectiveness of literary devices. I have offered hypotheses about how other devices create aesthetic states. Can alternatives to my hypotheses be generated, and tests of these competing hypotheses be devised? The present account differs from many contemporary views in claiming that aesthetic experience is unipolar, and that negative reactions to art are not aesthetic responses. What empirical evidence could substantiate or refute that claim?

A century and a half after Fechner began it, the field of psychological aesthetics offers rich possibilities for creative researchers who address such questions.

## Data Availability

The original contributions presented in the study are included in the article/supplementary material, further inquiries can be directed to the corresponding author.

## Footnotes

1 Consider the complexity: When listening to music, we may understand what mood is intended without actually perceiving it in the object—the experience we characterize in the phrase “I know what they’re trying to do, but it’s not working.” At other times, the aesthetic effect is realized, and we ascribe the emotion to the music—“that song is so sad.” In this case we experience an R-emotion. However, it is also possible that the music may actually induce the emotion in us; by a kind of contagion, we have ourselves become sad, or excited, or angry. That would be an E-emotion and an unsatisfactory aesthetic experience, as when a tragic story leaves us depressed rather than moved. Aesthetically naïve listeners may misattribute to themselves an emotion that actually inheres in the object—may mistake an R-emotion for an E-emotion. That fact complicates research on how artworks make us feel.

2
www.humorstudies.org

3 Schoeller (2015), Schoeller et al. (2024a) provide extensive empirical research on aesthetic chills.

4 This phenomenon is known as *rhythmic entrainment*, and Juslin (2013) regarded it as an evolved mechanism that can stimulate emotional bonding. This may be true, especially in a communal context such as a sing-along, but it is also a fundamental aesthetic device. The quality it imparts to a work probably falls into the category of hypnotic fascination—or so Ira Gershwin thought.

5
https://en.wikipedia.org/wiki/The_Outer_Limits_(1963_TV_series)

6 For example: https://www.youtube.com/watch?v=s6Uc4hrCyKw

7
https://www.youtube.com/@robertmccrae7740/playlists
